# Salvage of Iatrogenic Sciatic Nerve Injury Caused by Operatively Treated Acetabular Fractures: Two Cases and Literature Review

**DOI:** 10.1111/os.14153

**Published:** 2024-06-25

**Authors:** Peng Zhang, Fulin Tao, Wenhao Song, Shuai Wu, Dawei Wang, Dongsheng Zhou, Fanxiao Liu

**Affiliations:** ^1^ Department of Orthopaedics Shandong Provincial Hospital Affiliated to Shandong First Medical University Jinan China

**Keywords:** Acetabular fractures, Electromyography, Iatrogenic sciatic nerve injury, Magnetic resonance imaging, Open reduction and internal fixation

## Abstract

**Background:**

While sciatic nerve injury has been described as a complication of acetabular fractures, iatrogenic nerve injury remains sparsely reported. This study aims to assess iatrogenic sciatic nerve injuries occurring during acetabular fracture surgery, tracking their neurological recovery and clinical outcomes, and investigating any correlation between recovery and the severity of neurologic injury to facilitate physicians in providing prediction of prognosis.

**Case Presentation:**

We present two cases of male patients, aged 56 and 22, who developed sciatic palsy due to iatrogenic nerve injury during acetabular fracture surgery. Iatrogenic sciatic nerve injury resulted from operatively treated acetabular fractures. Surgical exploration, involving internal fixation removal and nerve decompression, successfully alleviated symptoms in both cases postoperatively. At the latest follow‐up, one patient achieved full recovery with excellent function, while the other exhibited residual deficits at the L5/S1 root level along with minimal pain.

**Conclusion:**

Sciatic nerve injury likely stemmed from reduction techniques and internal fixation procedures for the posterior column, particularly when performed with the hip flexed, thereby placing tension on the sciatic nerve. Our case reports underscore the significance of liberal utilization of electrophysiologic examinations and intraoperative monitoring for the prediction of prognosis. Surgical exploration, encompassing internal fixation removal and nerve decompression, represents an effective intervention for resolving sciatic palsy, encompassing both sensory neuropathy and motor symptoms.

## Graphical abstract

(A) The anteroposterior radiograph reveals the placement of both screws from anterior to posterior; (B) the computed tomography scan illustrates the anterior‐to‐posterior placement of both screws, with their ends impinging on the sciatic nerve, and the sciatic nerve compression site is adjacent to the two screws and the surrounding soft tissue proliferates (arrows); (C) intraoperative photograph depicting the anterior‐to‐posterior positioning of two screws, with their ends causing slight damage to the sciatic nerve; and (D) postoperative anteroposterior radiograph demonstrating satisfactory reduction and internal fixation following revision surgery.

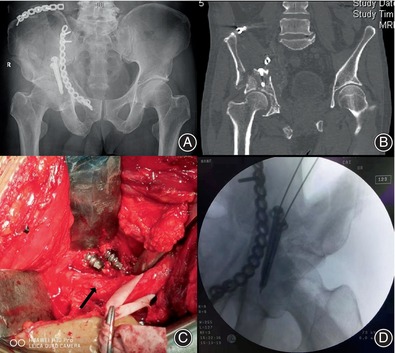

## Introduction

Traumatic sciatic nerve injury emerges as a particularly concerning complication of acetabular fractures due to the intricate anatomical relationships in the local region.^1^, ^2^ Due to special anatomical relationships between the sciatic nerve and the posterior wall of the acetabulum, the sciatic nerve injury is often accompanied by a displaced acetabular fracture or dislocated femoral head.^3^, ^4^ The incidence of sciatic nerve injury associated with fractures of the acetabulum ranges from 10 to 30%.^5^ Incorrect diagnosis and treatment may have a severe impact on the function of the lower extremity.^6^ Extensive studies underscore the sciatic nerve's comprehensive innervation of nearly all areas below the knee, highlighting its indispensable role in lower limb mobility and sensation.^7^, ^8^, ^9^, ^10^, ^11^, ^12^

As the common trunk of the sciatic nerve traverses the upper part of its trajectory, it courses deep within the gluteus maximus muscle, resting upon the posterior surface of the ischium. The unique anatomical relationship between the sciatic nerve and the posterior wall of the acetabulum predisposes the nerve to injury, often in conjunction with displaced acetabular fractures or dislocated femoral heads. The misdiagnosis or inadequate treatment of sciatic nerve injuries can profoundly impair lower limb function, underscoring the critical importance of accurate diagnosis and appropriate management.

Sciatic nerve injury, whether arising from iatrogenic or traumatic causes, poses considerable risks to patients. Understanding the disparities between these two types of injuries is crucial for effective management and prevention strategies. Recent studies have shed light on the pathophysiology, diagnosis, and management of sciatic nerve injury. Research has elucidated the mechanisms underlying nerve damage and explored innovative therapeutic approaches to enhance nerve regeneration and functional recovery.^13^, ^14^ However, despite these advancements, the severity of sciatic nerve injury and its long‐term consequences remain significant concerns. Sciatic nerve injury can lead to debilitating consequences, including motor and sensory deficits, chronic pain, and impaired quality of life. Additionally, severe cases may result in permanent disability, necessitating extensive rehabilitation and long‐term care. The profound impact of sciatic nerve injury underscores the urgent need for preventive measures and optimized treatment strategies. Iatrogenic injuries occur as a result of medical interventions, such as surgical procedures or injections, while traumatic injuries result from external forces or accidents. Understanding the distinct etiologies and risk factors associated with each type of injury is crucial for accurate diagnosis and appropriate intervention. Iatrogenic sciatic nerve injury represents a preventable complication of medical procedures. Vigilance and adherence to best practices can significantly reduce the incidence of iatrogenic injuries.^15^ Moreover, raising awareness among healthcare providers regarding the potential risks of certain interventions is imperative for patient safety and optimal outcomes.

The co‐occurrence of acetabular fractures and sciatic nerve injuries exhibits an incidence ranging from 3.3% to 33%.^3^, ^5^, ^16^ Electromyography serves as a valuable tool in distinguishing sciatic neuropathy from other leg‐related conditions such as plexopathy, radiculopathy, or disorders affecting the peroneal or tibial nerves.

While preoperative clinical examinations play a pivotal role in identifying sciatic nerve injuries, intraoperative confirmation of nerve abnormalities by surgeons expedites diagnosis and treatment.^17^, ^18^, ^19^ Existing literature suggests that a substantial proportion of traumatic sciatic nerve injuries exhibit spontaneous recovery with favorable outcomes.^20^, ^21^ A retrospective analysis^9^ conducted by our team explored patient characteristics, clinical manifestations, and management strategies pertaining to traumatic sciatic nerve injuries associated with acetabular fractures. The study evaluated the nuances of neurological recovery from such injuries and identified factors potentially influencing recovery. It was observed that all traumatic sciatic nerve injuries involved the posterior wall or posterior column of the acetabulum, with the majority of patients presenting posterior dislocation of the hip joint. Notably, preoperative damage to the common peroneal nerve division tended to be more severe than that affecting the tibial nerve division.^9^

While sciatic nerve injury has been documented as a complication of acetabular fractures, reports of iatrogenic nerve injury remain scarce. The present case study endeavors to scrutinize iatrogenic sciatic nerve injuries arising from operative interventions in acetabular fracture surgery. The study aims to monitor neurological recovery and outcomes in such cases, delving into any potential correlations between recovery and the severity of neurologic injury (whether complete or incomplete, motor/sensory/both). Insights gleaned from this study hold promise in enabling physicians to furnish patients with informed prognostic data regarding their recovery prospects.

## Case Presentation

### Case 1

A 56‐year‐old male presented to our outpatient for clinic seeking further management following surgery for a double column fracture performed elsewhere. He had experienced paralysis of his right lower extremity subsequent to pelvic surgery conducted 4 months earlier. The initial surgical approach involved K‐L approach for exposure of the posterior aspect of the hip joint, with the posterior column fragment being reduced and stabilized using two compression screws inserted from posterior to anterior.

**FIGURE 1:**
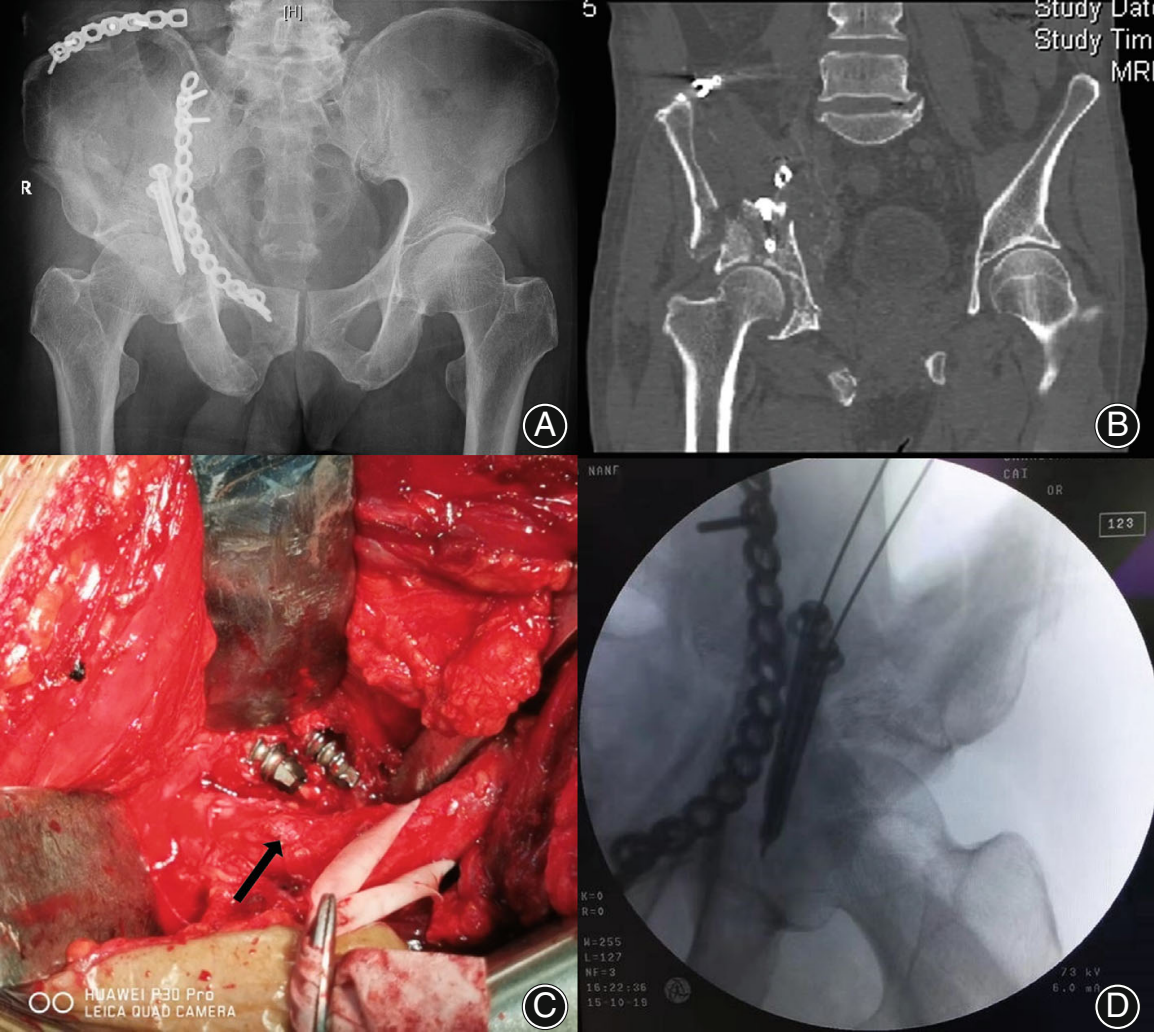
(A) The anteroposterior radiograph reveals the placement of both screws from anterior to posterior. (B) The computed tomography scan illustrates the anterior‐to‐posterior placement of both screws, with their ends impinging on the sciatic nerve, and the sciatic nerve compression site is adjacent to the two screws and the surrounding soft tissue proliferates (arrows). (C) Intraoperative photograph depicting the anterior‐to‐posterior positioning of two screws, with their ends causing slight damage to the sciatic nerve. (D) Postoperative anteroposterior radiograph demonstrating satisfactory reduction and internal fixation following revision surgery.

Unfortunately, despite the surgical intervention, the patient continued to experience paralysis of his right lower extremity along with persistent pain. A computed tomography (CT) scan was subsequently performed, revealing an overlength implant (Figure 1A,B). Clinical examination findings strongly suggested that the ongoing nerve injury was associated with the previous acetabular fracture surgery, prompting the decision for a revision procedure.

The patient underwent open reduction and nerve exploration, with the previously utilized standard K‐L approach selected for access. Upon exposure of the sciatic nerve, it was observed that two 3.5 mm screws had penetrated the bone cortex at the ischial tuberosity site, providing additional stability. However, the length of these screws was sufficient to cause compression against the sciatic nerve (Figure 1C). Recognizing the implant‐associated sciatic nerve injury, removal of the implant and replacement with shorter screws was performed to mitigate further nerve damage.

Digital electromyography (EMG) and nerve conduction recording were performed using an electromyography system before and after surgery, and the neuroelectrophysiological detection was performed during the surgical process. Follow‐up radiographs demonstrated the restoration of the posterior column surface (Figure 1D). Notably, no early complications such as infection or loss of reduction were observed following the revision surgery. The patient was able to achieve full weight‐bearing ambulation without difficulty after 3 months. Subsequent assessments at the 1‐year mark revealed pain‐free ambulation and excellent functional outcomes with the Harris hip score of 91 points, including pain (40 points), function (43 points), deformity (4 points), and range of motion (5 points).

### Case 2

A 22‐year‐old male presented to our outpatient clinic with complaints of excruciating pain in his left leg, accompanied by insomnia throughout the night. Despite a previous diagnosis of a normal sciatic nerve just 1 month ago, he experienced a significant deterioration in his left lower extremity function following acetabular surgery performed 2 weeks prior. A month and a half ago, the patient was treated in a local hospital because of hip pain and limited activity caused by a car accident. The patient was diagnosed as acetabular posterior column with posterior wall fracture. The local hospital adopted the posterior K‐L approach and greater trochanter osteotomy to treat the acetabular fracture with plate and screws. Postoperative pain occurred in the left lower limb after first surgery.

Upon physical examination, neurological assessment revealed pain and proprioceptive deficits in the left lower extremity, albeit without withdrawal reflex deficits. Vascular examination yielded normal findings. A diagnosis of sciatic nerve paralysis subsequent to acetabular fracture repair with bone plating was established in the left lower limb. Initial management involved conservative treatment with physiotherapy in a local hospital, but the patient exhibited poor recovery within 2 weeks.

**FIGURE 2:**
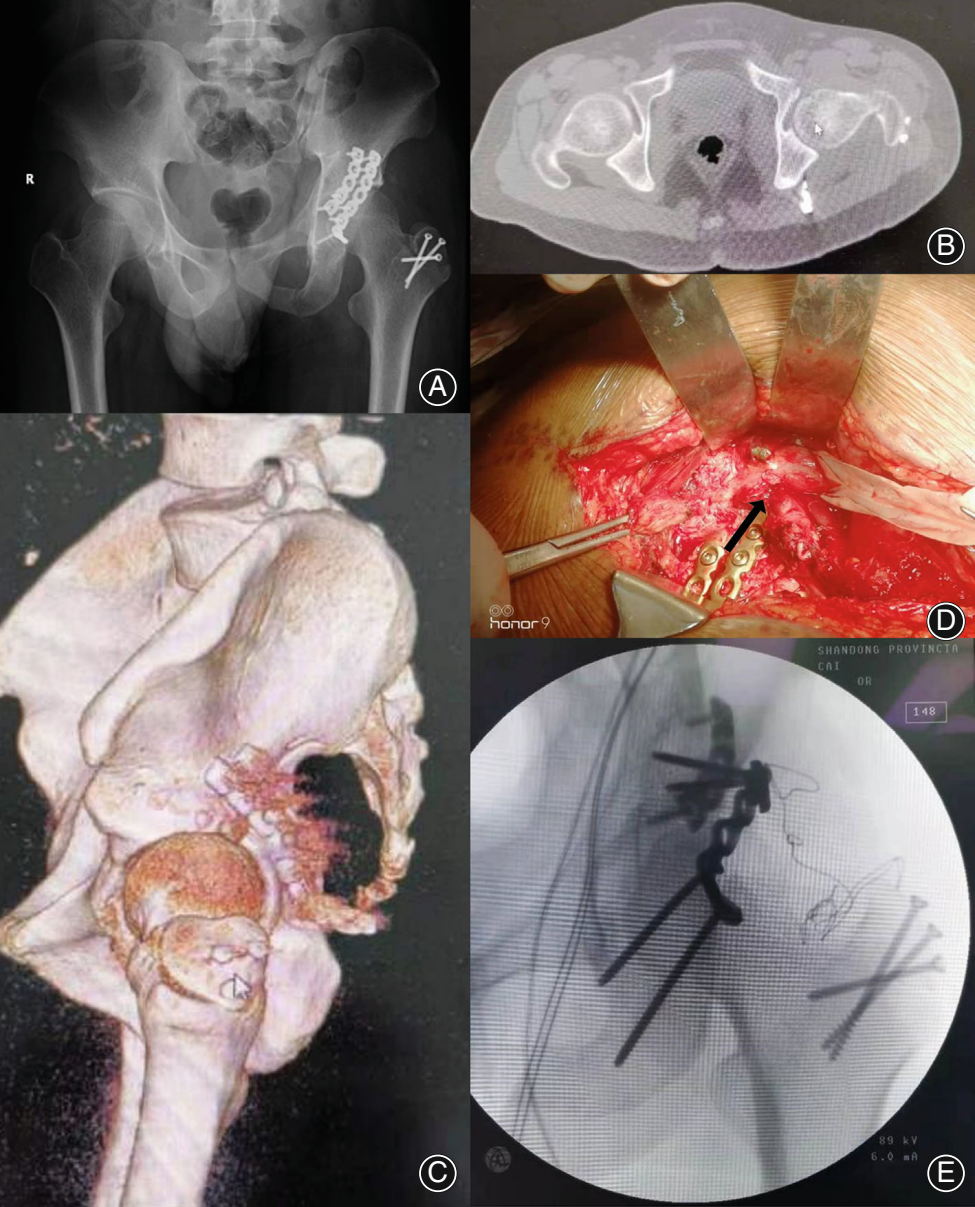
(A) The anteroposterior radiograph displays an implant on the posterior column of the acetabulum with a unique shape and abnormal positioning. (B, C) Computed tomography scans depict the implant situated directly within the region of the sciatic nerve. (D) Intraoperative view showing partial piercing and tight compression of the sciatic nerve under the distal end of the misplaced implant, and the sciatic nerve compression site is adjacent to the two plates and the surrounding soft tissue proliferates (arrows). (E) Postoperative anteroposterior radiograph indicating successful reduction and internal fixation achieved during revision surgery.

Radiographic and CT evaluations unveiled an implant situated on the posterior column of the acetabulum, displaying an unconventional shape and abnormal positioning (Figure 2A–C). These findings correlated directly with the patient's preoperative clinical symptoms, underscoring the need for surgical intervention.

In preparation for surgery, the patient was positioned in the side decubitus position, and the original posterior approach was employed to expose the sciatic nerve at the posterior aspect of the hip joint (Figure 2D). Surgical exploration revealed interposed fibrous tissue around the sacrotuberal ligament, necessitating meticulous dissection. Anatomical reduction of the posterior column of the acetabulum was confirmed *via* direct visualization. Subsequently, two 3.5 mm reconstruction plates were strategically positioned as buttress plates to ensure stability. However, it was noted that the sciatic nerve was partially pierced and tightly compressed beneath the distal end of the misplaced implant (Figure 2E). Prompt action was taken to address the implant‐associated sciatic nerve injury, involving immediate implant removal and repositioning to a safer, essentially “free‐floating” position.

Utilizing a digital electromyography (EMG) system, nerve conduction recordings were conducted intraoperatively to assess nerve function. The revision surgery and pharmacological interventions including painkillers (Tramadol) and nutritional nerve drugs (Mecobalamin) provided immediate relief from paralysis, particularly alleviating the patient's pain. Despite these interventions, the patient continued to experience debilitating deficits, notably loss of strength, even after undergoing intensive physiotherapy for duration of 3 months. Subsequent assessments at the 1‐year mark revealed pain‐free ambulation and good functional outcomes with the Harris hip score of 85 points, including pain (36 points), function (40 points), deformity (4 points), and range of motion (5 points).

## Discussion

Although the sciatic nerve, being the largest peripheral nerve, is inherently well‐protected due to its deep location near bone and substantial muscle mass, sciatic nerve palsy associated with acetabular fractures can arise from various sources, including trauma‐related damage, iatrogenic injury during reconstructive surgery, or postoperative complications.^17^ Iatrogenic causes encompass intraoperative positioning, retractor placement, instrumentation, surgical approach, erroneous anatomical identification, or abnormal implants.^17^, ^22^, ^23^ Late complications leading to sciatic nerve injury may include wear debris, implant migration, hematoma formation, capsule or muscle scarring, and heterotopic ossification resulting from the use of dilation approaches.

Distinguishing iatrogenic lesions from traumatic injuries during surgical treatment has been challenging historically. The posterior approach is commonly utilized for addressing posterior wall and column acetabular fractures, where sciatic nerve damage may directly result from implant injury or subsequent scarring involving the nerve.^24^ However, such cases are exceedingly rare, with only a limited number reported in the literature (Table S1).

A meticulous medical history is imperative to elucidate the temporal association between symptom onset and acetabular injury or reconstruction surgery, as well as any potential degenerative diseases of the lumbosacral region. Patients suffer from a spectrum of sensory and motor symptoms. Various imaging modalities, including plain radiographs, CT, MRI, ultrasonography, and electromyography, can aid in localizing nerve injuries anatomically.^25^ However, MRI may be impractical due to metal artifacts postoperatively. Electromyography has demonstrated utility in identifying the anatomical site of nerve damage and determining the nature of the injury (neuropraxia, axonotmesis, or neurotmesis).^26^

We present two cases wherein patients developed progressive sciatic neuropathy with radicular pain subsequent to nerve compression. In both instances, the placement of screws or plates in the affected region resulted in direct sciatic nerve injury during posterior exposure and implant placement. The neurological injury may have developed due to retraction during surgical exploration. An improper implantation technique may be another cause of injury. Retraction injury needs follow‐up, on the other hand, improper implant size needs revision surgery. Surgical exploration, involving the removal of internal fixation and nerve decompression, successfully alleviated symptoms in the immediate postoperative period. Specialized Hoffman‐type retractors designed for the lesser sciatic notch were employed to safeguard the nerve during surgery. Furthermore, meticulous attention was paid to maintaining the obturatorinternus muscle and tendons between the nerve and the retractors during exposure to minimize nerve injury risk.

Treatment methods for nerve injury encompass conservative management and surgical intervention.^27^ In cases of severe paralysis, early operative neurolysis is crucial for restoring sciatic nerve function. Intraoperative fluoroscopy may be employed to ensure safe placement of instruments or implants. Somatosensory evoked potentials (SSEP) and spontaneous electromyography can serve as valuable intraoperative indicators of potential nerve damage.^28^ The outcome of surgical decompression appears to hinge onvarious preoperative factors, with sensory paralysis exhibiting better outcomes than motor paralysis.^29^

Our case studies demonstrate the efficacy of sciatic nerve neurolysis following fixator removal for iatrogenic sciatic nerve injury in acetabular fractures. We delineate the unique aspects of the injury based on the initial surgical procedure performed and subsequent clinical and electrophysiologic evaluations. Preventive measures are involved in avoiding iatrogenic stretch damage to the nerve by maintaining hip extension and knee flexion during fracture fixation. Careful positioning of retractors and minimizing extensive posterior retraction with the hip in flexion are essential precautions. Our findings underscore the critical importance of incorporating electrophysiologic examination and intraoperative monitoring into clinical practice, while emphasizing the effectiveness of surgical exploration, internal fixation removal, and nerve decompression in resolving sciatic palsy, encompassing both sensory neuropathy and motor symptoms.

The recovery of the sciatic nerve is intricately linked to the severity of nerve damage. Complete injuries, resulting in total loss of motor and sensory function, often lead to prolonged rehabilitation and limited recovery. Conversely, incomplete injuries, retaining partial function, generally have a more favorable prognosis with improved recovery potential. Motor deficits, such as muscle weakness or paralysis, and sensory deficits, including numbness or tingling, presented unique challenges for rehabilitation. In cases where both motor and sensory functions are affected, recovery may be more complex and require tailored interventions. Understanding this correlation is crucial for guiding treatment strategies and optimizing outcomes in individuals with sciatic nerve injuries.

The differences in prognosis observed between the two cases of iatrogenic sciatic nerve injury following acetabular fracture surgery warrant discussion. Despite both patients undergoing revision surgery to alleviate symptoms, Case 1 achieved full recovery with excellent functional outcomes, while Case 2 experienced persistent deficits, particularly in terms of strength, even after intensive physiotherapy. Several factors may contribute to these disparate prognoses. First, the nature and extent of nerve injury differed between the two cases. In Case 1, the sciatic nerve was compressed by overlength screws, leading to immediate recognition and prompt surgical intervention. Conversely, in Case 2, the nerve was partially pierced and tightly compressed beneath a misplaced implant, potentially resulting in more severe and prolonged nerve damage. Furthermore, the timing of intervention may have influenced outcomes. Case 1 underwent revision surgery relatively promptly after the onset of symptoms, whereas Case 2 experienced a delay in surgical intervention, with conservative management initially attempted. Early recognition and intervention are crucial for optimizing nerve recovery and minimizing long‐term sequelae. Additionally, individual patient factors, such as age, overall health status, and the presence of comorbidities, may have influenced prognosis. Case 1, being older and potentially having less comorbidity, may have had a more favorable response to surgical intervention compared to the younger and potentially healthier individual in Case 2. In summary, the differences in prognosis between the two cases of iatrogenic sciatic nerve injury likely stem from variations in the nature of nerve damage, timing of intervention, individual patient factors, and intraoperative management techniques. Further research and clinical experience are needed to elucidate optimal strategies for managing and optimizing outcomes in such cases.

## Conclusion

In summary, our case reports underscore that sciatic nerve injury likely resulted from reduction techniques and internal fixation for the posterior column performed with the hip flexed, thereby subjecting the sciatic nerve to tension, and underscore the critical importance of recognizing and addressing iatrogenic sciatic nerve injuries arising from reduction techniques and internal fixation procedures for acetabular fractures. The severity of nerve injury, whether complete or incomplete, motor or sensory, significantly impacts recovery outcomes. While prompt surgical exploration, internal fixation removal, and nerve decompression represent effective interventions, understanding the correlation between injury severity and recovery is crucial for optimizing patient outcomes. Moreover, liberal employment of electrophysiologic examination and intraoperative monitoring is essential for early detection and management of nerve injuries during surgery.

## Consent for Publication

We confirm that the work described has not been published before; that it is not under consideration for publication elsewhere; that its publication has been approved by all the authors and that its publication has been approved by the responsible authorities at the institution where the work was carried out. A statement regarding ethics approval and written informed consent was obtained from the patients for publication of this case report and accompanying materials.

## Conflict of Interest Statement

The authors report no conflicts of interest in this work.

## Ethics Statement

Written informed consent for publication was obtained from the patients.

## Author Contributions

ZP and LFX wrote the manuscript including the literature review. ZP and LFX had substantial contributions to conception and design. ZP and LFX had been involved in revising the manuscript critically. All authors have read and approved the final manuscript and greed both to be personally accountable for the authors’ own contributions and to ensure that questions related to the accuracy or integrity of any part of the work, even ones in which the author was not personally involved, are appropriately investigated, resolved, and the resolution documented in the literature.

## Supporting information


**Table S1.** Main characteristics and data of sciatic nerve injury reported in the literature.

## Data Availability

We confirm that all the supporting data mentioned in this manuscript can be provided.
