# Supplementary material for: Salvage of Iatrogenic Sciatic Nerve Injury Caused by Operatively Treated Acetabular Fractures: Two Cases and Literature Review
Source: Orthop Surg. 2024 Jun 25;16(8):2100–6. doi: 10.1111/os.14153 (PMC11293912; doi:10.1111/os.14153)
Supplement: Supplementary file 1 — Table S1. Main characteristics and data of sciatic nerve injury reported in the literature. [file OS-16-2100-s001.docx]

**Supplementary Table 1.** Main characteristics and data of sciatic nerve injury reported in the literature

| **Study, year** | **Country** | **Type** | **Included cases** | **Cases (follow-up)** | **Fracture type** | **Approach** | **Cases with post-traumatic/**  **iatrogenic sciatic nerve injury** | **Recovery in post-traumatic/iatrogenic injury** | **Final follow-up (months, range)** | **Time until  complete recovery (months)** |
| --- | --- | --- | --- | --- | --- | --- | --- | --- | --- | --- |
| Al Adawy,  et al. 2020[1] | Egypt | R | 38 | 38 | Any type | Ilioinguinal, modifed Stoppa | 0/3 | -/3 complete | 18 | 6-12 |
| Almeida,  et al. 2011[2] | Brasil | P | 76 | 76 | Any type | K-L, K-L + IL, IL, iliofemoral | 7/3 | 5 complete,2 none/3 complete | 4.9 (4-9)yrs/- | NA |
| Ebraheim,  et al. 2007 [3] | USA | R | 32 | 32 | Post wall | K–L | 2/0 | 2 complete/- | 43 (24–70) | NA |
| Faizi,  et al. 2014 [4] | Malaysia | R | 30 | 30 | NA | K–L | 0/3 | -/1 complete, 2 none | 12 | 10 |
| Giordano,  et al. 2009 [5] | Brasil | P | 35 | 35 | Transverse with post wall | K–L | 3/0 | 3 complete/- | 12 | NA |
| Gultac,  et al. 2019 [6] | Turkey | R | 21 | 21 | Post wall | K–L | 1/0 | 1 complete/- | 24 (12-60) | 8 |
| Gupta,  et al. 2009 [7] | India | R | 68 | 63 | Any type | K-L, IL, iliofemoral, triradiate, combined | 0/2 | -/1 complete, 1 partial | 52.94 (37–96) | 3 |
| Gupta,  et al. 2015 [8] | India | R | 64 | 52 | Any type | KL, IL, triradiate, THR (Moore's) | 5/1 | 2 complete, 1 partial,2 none/1 partial | 60.3 (26–136) | NA |
| Gupta, et al. 2017 [9] | India | R | 25 | 25 | Post wall/column/transverse | K–L + trochanteric osteotomy | 2/1 | 2 complete/ 1 complete | 6–15 | 12 |
| Gupta,  et al. 2018 [10] | India | R | 6 | 6 | Post wall | K–L | 1/0 | 1 complete/- | 47.66 (43– 57) | 24 |
| Harnroongroj,  et al. 2013 [11] | Thailand | R | 21 | 21 | Post wall | K–L | 0/3 | -/3 none | 36 | NA |
| Iqbal,  et al. 2016 [12] | Pakistan | P | 60 | 50 | Any type | K-L, IL, triradiate | 6/0 | 4 complete, 2 none/- | 24 | 12 |
| Kim,  et al. 2011 [13] | Korea | P | 47 | 33 | Any type with post wall | K–L | 4/0 | 2 complete, 2 partial/- | 24 | NA |
| Kim,  et al. 2015 [14] | Korea | R | 22 | 20 | Any type | Modifed Stoppa, K-L | 3/0 | 2 complete,1 none/- | 30 (24—341) | NA |
| Liu,  et al. 2010 [15] | China | R | 19 | 19 | Any type | K-L, IL  or combined | 2/0 | 2 complete/- | 58.5 (25–103) | 2 |
| Magu,  et al. 2014 [16] | India | R | 25 | 25 | Post wall | K–L | 0/1 | -/1 complete | 60 | 15 |
| Malhotra,  et al. 2019 [17] | India | P | 18 | 18 | NA | K–L | 1/1 | 1 none/1 complete | 57.6 (48–70) | 4 |
| Masse,  et al. 2013 [18] | Italy | R | 31 | 31 | T-type, isolated transverse, or with post wall | K–L + trochanteric osteotomy | 2/0 | 2 partial/- | 43(24–87) | NA |
| Paksoy,  et al. 2019 [19] | Turkey | R | 74 | 35 | Any type | IL or modifed Stoppa ± K-L | 2/0 | 1 complete, 1 partial/- | 21.3 (12–47) | NA |
| Wang,  et al. 2018 [20] | China | R | 21 | 21 | Pipkin IV (femoral head plus post  wall) | K–L | 3/0 | 3 partial/- | 49 (36–76) | NA |

R, respective; P, prospective; NA, not available; K-L, Kocher-Langenbeck; THR, total hip replacement

**References:**

1. Al Adawy AS, Aziz AHA, El Sherief FA, et al. Modified Stoppa as an alternative surgical approach for fixation of anterior fracture acetabulum: a randomized control clinical trial. J Orthop Surg. 2020;15:154. <https://doi.org/10.1186/s13018-020-01660-3>.

2. de Almeida AGI, Garrido CA, Vaz Amaral LE, et al. Prospective study on seventy-six cases of fractured acetabulum with surgical treatment. Rev Bras Ortop. 2011;46(5):520–5. https://doi.org/10.1016/S2255-4971(15)30406-7.

3. Ebraheim NA, Patil V, Liu J, et al. Reconstruction of comminuted posterior wall fractures using the buttress technique: a review of 32 fractures. Int Orthop. 2007;31(5):671–5. https://doi.org/10.1007/s00264-006-0246-0.

4. Anizar-Faizi A, Hisam A, Sudhagar K, et al. Outcome of surgical treatment for displaced acetabular fractures. Malays Orthop J. 2014;8(3):1–6. <https://doi.org/10.5704/MOJ.1411.001>.

5. Giordano V, do Amaral NP, Pallottino A, et al. Operative treatment of transverse acetabular fractures: is it really necessary to fix both columns? Int J Med Sci. 2009;6(4):192–9. https://doi.org/10.7150/ijms.6.192.

6. Gültaç E, İltar S, Özmeriç A, et al. Surgical treatment of acetabulum posterior wall fractures: comparison between undercountering and marginal impaction reconstruction method with odd methods. J Clin Orthop Trauma. 2019;10(5):900–3. https://doi.org/10.1016/j.jcot.2019.01.023.

7. Gupta RK, Singh H, Dev B, et al. Results of operative treatment of acetabular fractures from the Third World—how local factors affect the outcome. Int Orthop. 2009;33(2):347–52. https://doi.org/10.1007/s00264-007-0461-3.

8. Gupta RK, Jindal N, Pruthi M. Acetabular fractures labelled poor surgical choices: analysis of operative outcome. J Clin Orthop Trauma. 2015;6(2):94–100. https://doi.org/10.1016/j.jcot.2015.03.003.

9. Gupta S, Singh J, Virk JS. The role of trochanteric flip osteotomy in fixation of certain acetabular fractures. Chin J Traumatol. 2017;20(3):161–5. <https://doi.org/10.1016/j.cjtee.2016.11.006>.

10. Gupta S, Mittal N, Virk JS. Use of tricortical iliac crest strut autograft in comminuted posterior wall acetabular fractures: a case series. Chin J Traumatol. 2018;21(1):58–62. https://doi.org/10.1016/j.cjtee.2017.08.005.

11. Harnroongroj T, Riansuwan K, Sudjai N, et al. Posterior acetabular arc angle of unstable posterior hip fracture–dislocation. Int Orthop. 2013;37(12):2443–9. https://doi.org/10.1007/s00264-013-2090-3.

12. Iqbal F, Taufiq I, Najjad MKR, et al. Fucntional and radiological outcome of surgical management of acetabular fractures in tertiary care hospital. Hip Pelvis. 2016;28(4):217–24. https://doi.org/10.5371/hp.2016.28.4.217.

13. Kim HY, Yang DS, Park CK, et al. Modified Stoppa approach for surgical treatment of acetabular fracture. Clin Orthop Surg. 2015;7(1):29–38. <https://doi.org/10.4055/cios.2015.7.1.29>.

14. Kim HT, Ahn J-M, Hur J-O, et al. Reconstruction of acetabular posterior wall fractures. Clin Orthop Surg. 2011;3(2):114–20. <https://doi.org/10.4055/cios.2011.3.2.114>.

15. Liu X, Xu S, Zhang C, et al. Application of a shape-memory alloy internal fixator for treatment of acetabular fractures with a followup of two to nine years in China. Int Orthop. 2010;34(7):1033–40. <https://doi.org/10.1007/s00264-009-0867-1>.

16. Magu NK, Gogna P, Singh A, et al. Long term results after surgical management of posterior wall acetabular fractures. J Orthop Traumatol Off J Ital Soc Orthop Traumatol. 2014;15(3):173–9. <https://doi.org/10.1007/s10195-014-0297-8>.

17. Malhotra R, Gautam D. Acute total hip arthroplasty in acetabular fractures using modern porous metal cup. J Orthop Surg Hong Kong. 2019;27(2):1–7. https://doi.org/10.1177/2309499019855438.

18. Masse A, Aprato A, Rollero L, et al. Surgical dislocation technique for the treatment of acetabular fractures. Clin Orthop. 2013;471(12):4056–64. https://doi.org/10.1007/s11999-013-3228-8.

19. Paksoy AE, Topal M, Aydin A, Zencirli K, Kose A, Yildiz V. Outcomes of surgical management of acetabular fractures treated with anterior approaches. Eurasian J Med. 2019;51(3):257–61. <https://doi.org/10.5152/eurasianjmed.2019.0241>.

20. Wang S-X, Li B-H, Li J, et al. Middle-term follow-up results of Pipkin type IV femoral head fracture patients treated by reconstruction plate and bioabsorbable screws. Chin J Traumatol. 2018;21(3):170–5. <https://doi.org/10.1016/j.cjtee.2017.12.004>.
